# Exploring Perspectives of Health Care Professionals on AI in Palliative Care: Qualitative Interview Study

**DOI:** 10.2196/79514

**Published:** 2025-12-08

**Authors:** Osamah Ahmad, Stephen Mason, Sarah Stanley, Amara Callistus Nwosu

**Affiliations:** 1 Northern Care Alliance NHS Foundation Trust Greater Manchester United Kingdom; 2 Palliative Care Unit Dept of Cardiovascular and Metabolic Medicine University of Liverpool Liverpool United Kingdom; 3 Liverpool John Moores University Liverpool United Kingdom; 4 Marie Curie North West Liverpool United Kingdom; 5 Lancaster Medical School Lancaster University Lancaster United Kingdom

**Keywords:** artificial intelligence, AI, digital health, education, ethics, technology, end of life, innovation

## Abstract

**Background:**

The use of artificial intelligence (AI) methods in palliative care research is increasing. Most AI palliative care research involves the use of routinely collected data from electronic health records; however, there are few data on the views of palliative care health care professionals on the role of AI in practice. Determining the opinions of palliative care health care professionals on the potential uses of AI in palliative care will be useful for policymakers and practitioners to determine and inform the meaningful use of AI in palliative care practice.

**Objective:**

This study aimed to explore the views of palliative care health care professionals on the use of AI for the analysis of patient data in palliative care.

**Methods:**

This was a phenomenological study using qualitative semistructured interviews with palliative care health care professionals with a minimum of 1 year of clinical experience in a hospice in the North West of England. Data were analyzed using inductive thematic analysis.

**Results:**

We interviewed 6 palliative care professionals, including physicians, nurses, and occupational therapists. AI was viewed positively, although most participants had not used it in practice. None of the participants had received training in AI and stated that education in AI would be beneficial. Participants described the potential benefits of AI in palliative care, including the identification of people requiring palliative care interventions and the evaluation of patient experiences. Participants highlighted security and ethical concerns regarding AI related to data governance, efficacy, patient confidentiality, and consent issues.

**Conclusions:**

This study highlights the importance of staff perceptions of AI in palliative care. Our findings support the role of AI in enhancing care, addressing educational needs, and tackling trust, ethics, and governance issues. This study lays the groundwork for guidelines on AI implementation, urging further research on the methodological, ethical, and practical aspects of AI in palliative care.

## Introduction

### Background

Artificial intelligence (AI) is the science and engineering of creating “intelligent” machines (computers) through developed algorithms that replicate the ability of a machine to think and act like a human [1]. AI involves different methodologies (eg, machine learning, neural networks, deep learning, and natural language processing) that enable a machine to be trained to act autonomously, with or without human instruction [2]. AI has already demonstrated a significant impact in practice by facilitating the interpretation and analysis of large amounts of data contained within electronic health care datasets [3-5]. In health care, AI can facilitate the diagnosis of diseases, support clinical care delivery, and help individuals maintain their independence [6]. AI is increasingly used in palliative care (a discipline that provides holistic, person-centered support for people with life-limiting illness [7]). For example, AI-driven data analysis of electronic health records has been used to identify palliative care needs [8], support clinical documentation [9], identify quality indicators for end-of-life care [10], support symptom assessments [11], and estimate prognosis [12]. Despite the increased focus on palliative care AI, most current clinical AI tools are designed for nonpalliative care populations [8]. The lack of palliative care AI tools is a consequence of limited evidence in populations with serious illness, who often experience complex physical and psychosocial needs [13]. Palliative patients often experience significant morbidity, complex and multifaceted symptoms, and a high risk of mortality, whereas their families face grief and bereavement that extend beyond the patient’s death [13]. These unique dimensions of care introduce ethical, practical, and emotional considerations for AI, which are not as prominent in other medical and surgical specialties [14]. For example, the use of AI in prognostication may be more important in palliative care, where sensitive communication about life expectancy directly shapes care planning and emotional preparedness [12,15]. Similarly, the handling of data after death (ie, digital legacy and the rights of caregivers and families) raises questions that are less relevant in acute or curative clinical contexts [16]. Despite the growing body of research on AI in medicine, there is currently limited evidence that addresses these specific complexities within palliative care, which highlights the need for targeted research in this field [17]. Consequently, understanding the views of palliative care practitioners on AI is important to better understand the opportunities, challenges, and implementation issues associated with its use [18]. Therefore, a dedicated study of palliative health care professionals’ perspectives on AI is warranted to explore the specific issues affecting this cohort [17,19].

### Aim

This study aimed to explore palliative care professionals’ views on the use of AI for the analysis of patient data in palliative care.

## Methods

### Overview

This study involved inductive thematic analysis, a method of analyzing qualitative data in which themes emerge directly from the data without predefined codes or expectations. Inductive thematic analysis was chosen as it provided the researcher with the flexibility to explore participants’ perspectives on AI in palliative care [20]. The lead researcher (OA) was a final-year medical student (male) who was supervised by SM and ACN. OA received training and support to conduct the interviews. The study adhered to the COREQ (Consolidated Criteria for Reporting Qualitative Research) checklist [21].

### Study Setting

The study was conducted in a hospice in the North West of England, a specialized health care facility providing care for individuals in the advanced stages of a terminal illness or approaching the end of their lives. The hospice provides various services, including a 15-bed inpatient unit, day services, outpatient clinics, community outreach, patient and family support, and bereavement services.

### Sampling and Recruitment

Recruitment was conducted between April and May 2022. The study was introduced at a weekly hospice education meeting, giving potential participants the opportunity to ask questions and express their interest in participating. Study advertisements were placed around the hospice, and an email was sent to all staff outlining the inclusion and exclusion criteria for the study. The study material included information about the researcher (OA). Inclusion criteria were palliative care health care professionals working in the hospice with a minimum of 1-year of clinical experience during the study data collection period of April to May 2022.

### Data Collection

Over a period of 2 months, semistructured interviews were carried out with palliative care health care professionals. Participants received written information before the interview and provided written consent. Interviews were conducted face-to-face in a meeting room at the hospice. All the interviews were audio recorded using a digital voice recorder. The interviews lasted between 35 and 60 minutes, and the participants were informed that they could pause or discontinue the interview at any time. Each interview began with the researcher providing the participants with background information about the study and a definition of AI*.* The researcher conducted interviews using an interview guide, which was used to encourage structure across interviews but also facilitated flexibility by allowing participants to talk freely about their experiences (Multimedia Appendix 1). Open questions were used, and the interview schedule was adapted throughout the course of data collection to reflect the emergent themes and concepts (Textbox 1). Field notes and reflections were written throughout the interview process to help make sense of the data during the analysis phase.

**Examples of the interview questions.**

- What is your experience with artificial intelligence (AI) currently used within palliative care, if any?
- What do you see as the desired goal or outcome of using AI within palliative care?
- Have you ever been educated on the use of AI within palliative care?
- What are your views on introducing education programs for palliative care health care professionals on the use of AI within palliative care?
- AI in palliative care, like any new health care technology, may raise several safety concerns. Do you have any such concerns?
- AI in palliative care, like any new health care technology, may raise several security concerns. Do you have any such concerns?
- What are some important metrics that should be used when comparing AI tools to traditional tools in palliative care?
- Can you identify a task at work which you currently perform repeatedly, with little or no variations each time?
- What level of trust would you need to allow AI tools to perform this task for you?
- What are some ways this level of trust can be established?
- Is there anything else you’d like to tell me about this topic? If not, do you have any questions?

Interviews were transcribed verbatim by OA. Data were exported to Microsoft Word, and manual thematic analysis coding was used to systematically label the qualitative data extracts to identify patterns and themes. OA used the 6-step thematic analysis proposed by Braun and Clarke [22], which involves (1) familiarization with the data, (2) generation of initial codes, (3) development of initial themes, (4) reviewing themes, (5) defining and naming themes, and (6) writing up the analysis. We used inductive analysis, with line-by-line coding of participants’ interviews, to generate codes and organize the data into themes (Multimedia Appendix 2). Coding was carried out by OA. During the data collection phase, OA had regular supervisory meetings (with ACN and SM) to review data analysis, discuss initial findings, and evaluate data throughout the analysis process.

Six individuals contacted OA to consent to participate. None of the participants withdrew from the study. Interviews were analyzed iteratively. By the fourth interview, all key major themes (eg, “general openness toward AI” and “importance of human contact, empathy, and sympathy”) were identified. Additional interviews reinforced, rather than expanded upon, these categories. For example, the theme of “confidentiality concerns” emerged in the first interview and was reiterated consistently in interviews 2 to 6 without new subcategories. This redundancy indicated that thematic saturation had been reached with the sample. We determined that further interviews were unlikely to provide new codes, categories, or insights relevant to the research question. Therefore, a consensus was reached to stop data collection on completion of 6 interviews (ie, to stop recruitment of further participants) as the theoretical categories became saturated [23,24].

### Ethics Statement

The University of Liverpool ethics committee gave ethics approval for this work (reference number 8523). This study was approved by the hospice research governance group. All participants provided written informed consent to participate in this study. All participants were informed of their right to privacy and confidentiality. All participants were informed that their data would be anonymized and that no identifying information would be included in any works related to this study. No compensation was given to participants for participating in the study.

## Results

### Overview

We interviewed 6 palliative health care professionals, including 1 (17%) nurse, 2 (33%) occupational therapists, and 3 (50%) physicians. A total of 4 (67%) participants were female, and 2 (33%) were male. All interviews were conducted face-to-face and in person (Table 1).

**Figure 1:**
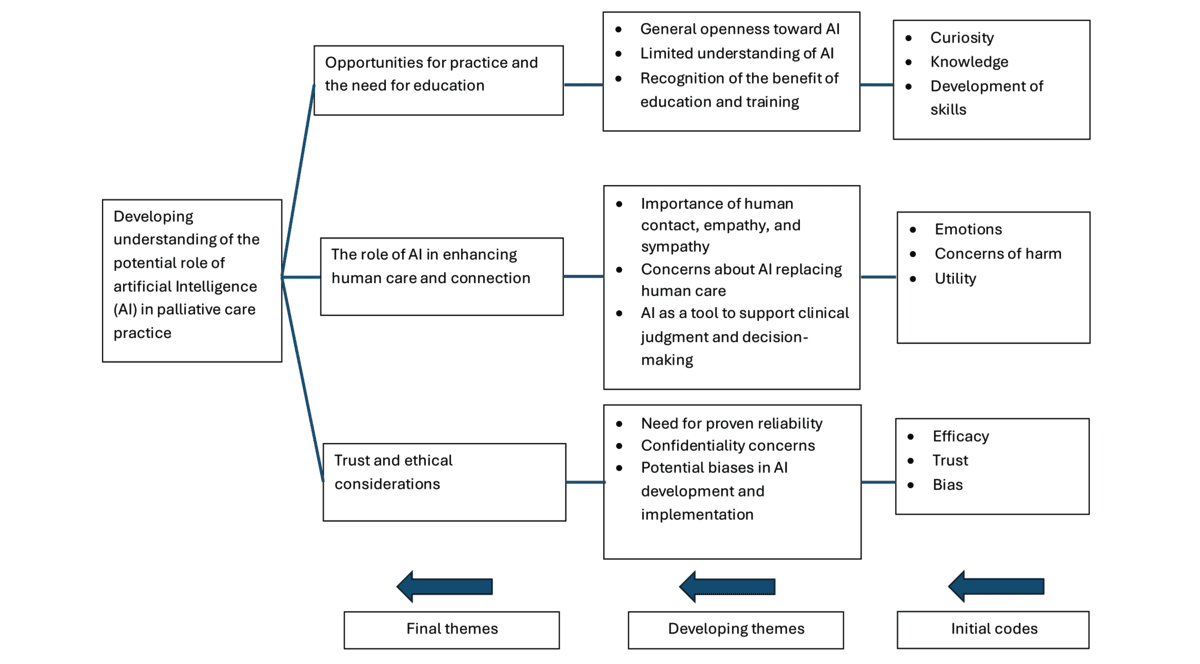
Thematic map showing the 3 main themes. AI: artificial intelligence.

Three themes were developed from the data: (1) opportunities for practice and the need for education, (2) enhancing human care and connection, and (3) trust and ethical considerations (Figure 1).

**Table 1:** Characteristics of the participants (N=6).

Characteristics			Participants, n (%)
**Profession**			
	Physician	3 (50)	
	Occupational therapist	2 (33)	
	Nurse	1 (17)	
**Sex**			
	Female	4 (67)	
	Male	2 (33)	
**Interview method**			
	In person	6 (100)	
	Online	0 (0)	

### Opportunities for Practice and the Need for Education

Participants highlighted their openness to consider using AI in palliative care practice. Specifically, participants described their views on how this technology could improve care delivery and efficiency for their patients. Participants spoke of how algorithm-driven health care is increasingly being used in other clinical specialties and how, similarly, AI can be used to improve palliative care:

I mean, I think obviously the future is, is going that way, isn’t it? You know, artificial intelligence is being developed at all areas.

Participant 3

Participants described the lack of AI use in palliative care compared to other medical and surgical specialties:

I suppose it’s kind of an alien concept, isn’t it? I think in terms of technology that we use day-to-day at the moment, we’re kind of behind the curve, I would say in, in the NHS [National Health Service], and certainly in this environment. So, I think it’s difficult to kind of visualise what that would be like in kind of day-to-day practice.

Participant 6

Participants spoke about how AI can potentially improve palliative care. The interviewees provided several examples of possible benefits, such as machine learning–based analyses of electronic health records to identify people needing palliative care, to improve data capture and analysis, and to inform personalized care recommendations:

I guess, you know, looking through the hospital records in the hospital. When I see a new patient, I’m manually looking back through the notes to try to find if they’ve had previous encounters with the palliative care team, how many times they’ve been in hospital, looking for all the medications to work out what medications they’re on and if any have been stopped.

Participant 3

A good thing would be if something could analyse the entire database every day and say, “look, these are the patients who are flagging up, particularly symptomatic,” or “are using lots of PRN [as required] medicines” or, you know, [if] certain keywords trigger [an] urgent review?

Participant 4

Participants highlighted that palliative care staff have a limited understanding of the different types of AI applications that may be used in clinical care. Participants expanded on this by describing how better staff education may improve their confidence in using AI in clinical practice:

I think for healthcare professionals, like for me, who’ve maybe not come across so much, or don’t understand it so much, it’s more about knowing about it, and understanding how we can help use it a bit better.

Participant 1

I think that’s only a positive thing. And, I think, to introduce education to help people understand even the basics of what AI is is really important, because I think until we know more about what it is and understand more about what it is, we can’t think of the ways in which we can improve care for the patient.

Participant 1

### Enhancing Human Care and Connection

Participants emphasized the importance of human contact, empathy, and sympathy in palliative care, expressing concerns about AI potentially replacing human care. This was illustrated by the comments of 1 participant:

I think in palliative care, you know, our strength is based on human connections.... I don’t mean to be like negative towards, kind of, AI technology and stuff, but I think, actually, you know, the majority of my day, when I’m speaking to patients, it’s about human connection.

Participant 6

Participants identified how AI systems could be potentially designed and modeled to dynamically learn from health care professionals and service users, with the objective to develop algorithms that are meaningful in helping them provide clinical care:

I think it should be modelled as if, you know, the algorithm behind it is trying to learn, and it’s asking you for confirmation. You know, so it’s saying, “this is what I’ve found,” you know, and you then have to decide whether this is useful, or “can you confirm this is correct?”, or something like that. It should be this kind of back and forth.

Participant 4

Participants described the potential that palliative care health care professionals can routinely use data, informed by AI, to support care and their clinical decision-making (eg, analysis of electronic health record data to identify patients with palliative care needs, automated transcription of consultations, and personalized treatment recommendations). Consequently, the interviewees discussed scenarios in which AI was used to improve and augment the clinical care provided by palliative care professionals. Participants said that AI could be used as a tool to support clinical judgment and decision-making while maintaining a patient-centered approach:

I guess if you use the program alongside, like, have a step-by-step approach, so you could use the program or the intelligence alongside, you know, a human in kind of partnership. And then gradually, you just have to learn that trust and become more used to it.

Participant 2

### Trust and Ethical Considerations

Participants highlighted the importance of health care professionals being confident that AI-driven clinical tools are trustworthy and reliable. The interviewees framed their opinions on the trustworthiness of AI in the context of their clinical responsibility of providing care for people with serious illness (who are often considered vulnerable). Several quotes from the participants illustrated the important role of research in generating evidence to inform meaningful AI use in clinical care:

It’d be track record and experience, wouldn’t it? So, you probably would want to have enough evidence to show that it made good decisions.

Participant 5

Participants stated that confidentiality was important, with several statements highlighting their concerns regarding data privacy and the security implications of using AI in clinical practice. They emphasized the importance of ensuring that patients’ data remain secure and protected when using AI to inform clinical decision-making in palliative care:

I suppose, privacy and data is the main concern as quite often it is in health care, isn’t it? And you know, making sure that data is secure and, you know, things like hacking aren’t an issue and people can’t access patients’ and relatives’ private data easily.

Participant 1

I think with any new technology to have to make sure it’s secure... And, you know, privacy concerns and breaches of confidentiality- So I think all those things are really important.

Participant 6

Participants discussed the risk of bias associated with AI analysis, which could create (and widen existing) inequalities in palliative care. In the interviews, the participants discussed the importance of developing strategies to reduce this risk of bias in AI algorithms and to explore public opinion about the role of AI in clinical practice. Participants described their belief that if AI should be used to improve holistic care for patients and those important to them. Further comments from the interviews highlighted the opinion that the use of AI in palliative care should be inclusive, unbiased, and ethical:

If our algorithms have been driven by developers in Silicon Valley in California and most of them are white, male and young, and the modelling has been tested on a particular set of individuals, which don’t have certain characteristics, which mean that certain people are not represented, you then might get a device or an algorithm which isn’t tailored for the needs of certain people.

Participant 4

### General Comments of Participants

Overall, AI was viewed positively by many participants, although many stated that they had not used it in practice. None of the participants had received training in AI, and all stated that they would have liked to have received education on this topic. Participants described their opinions on how data science can improve clinical care; potential ideas included the use of AI to identify people from electronic health records who need palliative care and to use data analytics to evaluate quality of care. Participants highlighted data privacy and ethical issues related to AI use in palliative care, including important related issues such as governance, confidentiality, and consent.

## Discussion

Concerning the use of AI in palliative care, it is important to consider the opportunities it offers, the educational needs of staff, the importance of human connections, and practical issues related to trust and ethics.

### Importance and Uniqueness of This Paper

This study provides an overview of the views of specialist palliative care health care professionals regarding AI in palliative care, which adds knowledge to the limited evidence base. Our qualitative approach facilitated an in-depth exploration of participants’ experiences, which captured their nuances and complexities. The evidence derived from this study improves knowledge about the views palliative care staff have about AI, which will shape its clinical use. Evidence demonstrates that improved staff involvement can improve the effectiveness and success of health care system interventions [25,26].

### Relation to Previous Work in This Area

In this study, the staff positively described potential opportunities in which AI could be used to support palliative care, with themes consistent with previous work conducted with generalist staff [27]. In our study, staff identified several hypothetical possibilities where AI could be positively used to improve their practice (eg, predictive modeling, text screening, symptom assessment, and communication), which are consistent with current developments of AI in palliative care [28].

Our findings align with previous research, which recommends that health care professionals receive formal education and training in AI [17,29]. Specifically, previous research advises palliative care education programs to include holistic overviews of AI technologies, ethical considerations, and case studies that highlight the real-world applications and challenges of AI in palliative care [30].

Our findings support previous work, which describes the importance of focusing on human connections in palliative care while ensuring that AI tools are meaningfully used to improve the experience of patients, caregivers, and staff [27]. Consistent with previous research, we highlight the potential problems and bias that may occur from using AI in palliative care, due to limited evidence of patient-centered outcomes measures in palliative care populations [14]. In our analysis, participants described the ethical challenges of using AI in palliative care (reporting themes of promoting transparency and accountability in AI systems, regular ethical review and continuous impact assessments, ensuring patient autonomy and informed consent, and safeguarding data privacy and security) [30]. The ethical themes reported in our study are similar to those in previous research [30,31], illustrating the need to incorporate ethical principles (such as the 4 principles) into decisions involving AI use in palliative care practice [32]. For example, autonomy (Do service users have a choice of how their data are collected, analyzed, and stored?), beneficence (Is AI used in the best interests of individuals or is the benefit mostly for groups, populations, or other stakeholders?), nonmaleficence (How can we ensure people are not harmed from AI in palliative care?), and justice (How can AI tools be used fairly and equitably, in vulnerable people, from different backgrounds, cultures, and geographies?) [33]. Furthermore, our results support the importance of integrating broader ethical theories into practice (eg, consequentialism, deontology, rights-based ethics, and virtue ethics) to provide clinicians and policymakers with a framework to make decisions on how to use emerging technologies in clinical practice [34]. These frameworks can be used to address uncertainty to help stakeholders’ (eg, health care professionals, managers, and policymakers) decision-making when considering how to responsibly use AI tools in palliative care [13]. Consistent with previous work, our findings reinforce the view that there is a risk that current AI applications lack engagement with the ethical complexities of real-world use in palliative care, which highlights questions about the adequacy of clinical practice safeguards [15,30].

### Limitations

This study is small, focused on one hospice in the North West of England, which means that the findings may not be generalizable to other palliative care settings (eg, home, community, hospital, and nursing homes). Although thematic saturation was achieved with 6 participants, this may have been influenced by the similarities of the participants. We acknowledge that a larger sample incorporating staff from a wider range of roles and professional backgrounds may have yielded more in-depth and diverse data. For example, this study lacks representation of some professional roles (eg, social work, spiritual care, pharmacy, and fundraising), which means there is a lack of data about how AI may impact wider specialist roles in the palliative care multidisciplinary team. Furthermore, our study did not include the perspectives of patients, caregivers, and other relevant stakeholders.

### Importance to Policy, Practice, and Research

Decision-makers should consider the perspectives of palliative care staff when developing and implementing AI tools for palliative care. When considering applications of palliative care AI, decision-makers should consider how these innovations will improve care, address human needs, and fulfill ethical and governance requirements. From an educational perspective, it is important that palliative care professionals are trained to safely and effectively use new technologies (eg, AI) in clinical practice. Activities to achieve this objective may include the development of training curricula for undergraduate and postgraduate students, including content on the opportunities and challenges of AI in health care, ethical considerations of its use, and governance issues [30,35].

Future research on palliative care AI should establish standardized reporting for studies, seek external validation, and consider ethical issues, which are needed to ensure that the clinical application of AI tools is safe, meaningful, and effective [15]. Future research should explore views (on palliative care AI) from different perspectives, including multidisciplinary teams, managers, patients, caregivers, and other relevant stakeholders. Researchers should involve interdisciplinary partnerships and collaboration to facilitate work across essential interconnected themes, such as design, computing, data analysis, ethics, and translational medicine [17,36,37].

### Conclusions

This study shows the importance of considering the views of palliative care professionals regarding the potential role of AI in clinical practice. Our findings demonstrate the importance of considering opportunities to meaningfully use AI to improve human-focused care, support staff education, and address practical issues related to trust, ethics, and governance. This study provides a foundation for developing guidelines for AI implementation in palliative care practice. Future research should examine methodological, ethical, and practical issues to ensure that AI best supports palliative care for people with serious illnesses.

## Appendix

Multimedia Appendix 1 Interview guide.

Multimedia Appendix 2 Generation of study themes.

## Abbreviations

AI: artificial intelligence
COREQ: Consolidated Criteria for Reporting Qualitative Research

## Data Availability

All data generated or analyzed during this study are included in this published article (and its supplementary information files).
